# Aberrant Alternative Splicing Is Another Hallmark of Cancer

**DOI:** 10.1155/2013/463786

**Published:** 2013-09-11

**Authors:** Michael Ladomery

**Affiliations:** Faculty of Health and Life Sciences, University of the West of England, Coldharbour Lane, Bristol BS16 1QY, UK

## Abstract

The vast majority of human genes are alternatively spliced. Not surprisingly, aberrant alternative splicing is increasingly linked to cancer. Splice isoforms often encode proteins that have distinct and even antagonistic properties. The abnormal expression of splice factors and splice factor kinases in cancer changes the alternative splicing of critically important pre-mRNAs. Aberrant alternative splicing should be added to the growing list of cancer hallmarks.

## 1. The Growing List of Cancer Hallmarks

In the year 2000, Doug Hanahan and Bob Weinberg published a paper in which they suggested that all cancers share six common features, or hallmarks [1]. They were self-sufficiency in relation to growth signals; insensitivity to growth inhibitory signals; limitless replicative potential; the ability to evade apoptosis; the ability to sustain angiogenesis; and lastly, the ability to invade tissues and metastasize. These hallmarks provided a useful framework with which to conceptualise cancer. The paper has been cited several thousands of times as a result. Despite the benefit of the six hallmarks concept, it became clear that other processes in cancer are also consistently altered. This led Hanahan and Weinberg to publish a follow-up review in 2011 in which they extended the cancer hallmarks to a list of ten. The four new hallmarks were the ability to evade the immune system, the presence of inflammation, the tendency towards genomic instability, and dysregulated metabolism [2]. The latter hallmark resonates with an observation made in the early 20th century by Otto Warburg, namely, that cancer cells are characterised by abnormal respiration and unusually high anaerobic metabolism [3]. This was called the “Warburg effect” and is generally thought to be linked to the fact that tumour cells need to adapt to hypoxic environments [4–6].

It is undoubtedly useful to think of common processes that apply to all cancers. The ten hallmarks suggest theoretical frameworks for research and therapy. However, several additional hallmarks could be added to the list, and there is also a significant amount of intersection between the ten hallmarks. Specific cancer-associated genes can also be involved in more than one hallmark. There is therefore a tension between the need to think systematically about cancer and the reality that cancer is a remarkably complex and heterogenous disease. 

## 2. Dysregulated Alternative Splicing Is Another Key Feature of Cancer

In cancer, genetic lesions arise in several forms including chromosomal rearrangements, point mutations, and gene amplifications. Genetic lesions often cause the activation of a proto-oncogene or the inactivation of a tumour suppressor gene. However, the very definition of oncogenes and tumour suppressors is not necessarily straightforward. Several proteins, in different contexts, can exhibit properties of both oncogenes and tumour suppressors. A classical example is the Wilms tumour suppressor gene *WT1*. The *WT1* gene was discovered in the early 1990s due to its association with a chromosome 11 deletion linked to WAGR syndrome (the Wilms tumour, aniridia, genitourinary problems, and mental retardation). Soon after its discovery, mouse knockout studies confirmed its involvement in urogenital development, and it was found to be inactivated in over 10% of Wilms tumours (nephroblastoma) consistent with being a classic tumour suppressor gene. However, over the years, it became apparent that *WT1* is involved in the development of several other organ systems and that it can also be overexpressed in many different types of cancer consistent with the properties of a proto-oncogene [7]. WT1 function is affected by alternative splicing altering its C-terminal zinc-finger domain, radically changing its DNA-binding properties. Thus, alternative splicing complicates the biological and biochemical activities of WT1. Not only must its expression be examined in cancer but the balance of its splice isoforms must also be measured. The same principle applies to many, perhaps most, widely studied cancer-associated genes—their function is significantly affected by alternative splicing.

In the 1970s and 1980s, it was thought that gene expression is regulated mainly at a transcriptional level. However, it is now clear that epigenetics and cotranscriptional and posttranscriptional processes are equally important. The discovery of splicing in the late 1970s was only the beginning of what was to become a very prominent field of research. The vast majority of human genes, perhaps over 94%, are alternatively spliced [8]. A cancer-associated gene can express splice isoforms that either favour or counteract the growth of cancer cells. For example, several regulators of apoptosis can express isoforms that are proapoptotic or antiapoptotic [9]. A frequently quoted example is Bcl-x, a member of the Bcl-2 family of proteins that regulates the permeability of the outer membrane of mitochondria. Whereas the Bcl-xS splice isoform is proapoptotic, the Bcl-xL isoform is antiapoptotic as it prevents the release of mitochondrial components that would lead to apoptosis.

Sometimes quite unexpected splice isoforms are discovered. For many years, VEGF-A was thought to be an exclusively pro-angiogenic growth factor. However, VEGF-A actually expresses splice isoforms that are anti-angiogenic. An alteration in the balance of VEGF-A splice isoforms can either promote or inhibit angiogenesis. The overexpression of the pro-angiogenic splice isoform of VEGF-A is consistently observed in solid tumours. By shifting the balance of expression in favour of the anti-angiogenic isoform of VEGF-A, angiogenesis and tumour growth can even be inhibited. Thus, the manipulation of VEGF-A alternative splicing might provide opportunities for novel therapeutic targets [10, 11].

**Figure 1:**
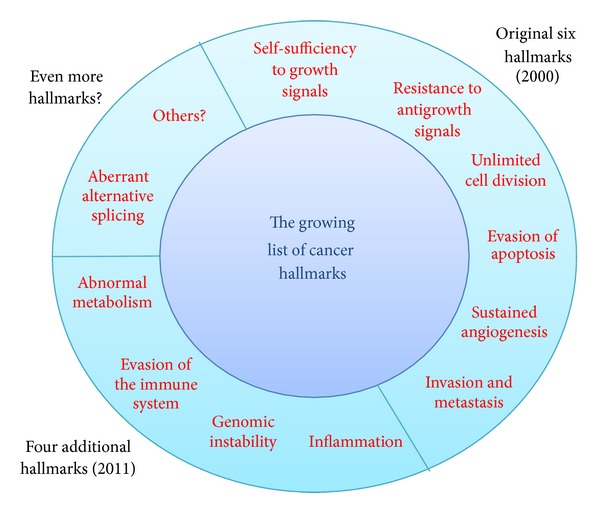
The original six hallmarks of cancer were proposed by Hanahan and Weinberg in 2000. Eleven years later, their list had grown to ten; but it could conceivably grow even further. An additional hallmark could be aberrant alternative splicing, in which the regulation of alternative splicing has gone astray systematically causing the inappropriate expression of multiple oncogenic splice isoforms.

Aberrant alternative splicing could also affect, systematically, an entire cancer-associated process, such as the epithelial to mesenchymal transition (EMT) [12]. In other words, the systematic and coordinated alteration of alternative splicing of several functionally linked pre-mRNAs could underpin specific processes in carcinogenesis. If this is the case, then dysregulated alternative splicing could surely consider itself a hallmark of cancer (Figure 1). But how might this work? Systematic changes in alternative splicing might be due to the inappropriate activation (or inactivation) of critically important splice factors or of the protein kinases and phosphatases that regulate their activity.

The fact that the expression of several RNA-binding proteins and splice factors is altered in cancer has been known for several years [13], but it is also clear that *specific* splice factors are particularly important in cancer. The splice factor SRSF1 (previously known as ASF/SF2), a member of the SR protein family (members of this family include RNA Recognition Motifs and serine-arginine rich domains), was described by Karni and colleagues as a proto-oncogene in 2007. Its overexpression transforms rodent fibroblasts allowing them to form sarcomas in nude mice [14]. SRSF1 also promotes the transformation of mammary epithelial cells by favouring the expression of antiapoptotic splice isoforms of BIM and BIN1 [15]. In fact, SRSF1 has several RNA targets in pre-mRNAs that are transcribed from genes implicated in cancer. Thus, the overexpression of SRSF1 could lead to a change in alternative splicing of several pre-mRNAs that, together, result in a phenotype that gives the tumour a growth advantage.

SRSF1 function and intracellular localization are regulated by protein kinases and phosphatases. The protein kinase SRPK1 phosphorylates SRSF1 in the cytoplasm favouring its nuclear accumulation. SRPK1 overexpression has also been observed in several cancers [16, 17], and so it is conceivable that its activation could also synergistically potentiate the oncogenic properties of SRSF1. This point is illustrated by the VEGF-A example. VEGF-A pre-mRNA is one of the targets of SRSF1. SRSF1 drives the expression of pro-angiogenic VEGF-A. Abnormally, high levels of SRPK1 cause the nuclear accumulation of SRSF1 in glomerular podocytes, leading to upregulation of pro-angiogenic VEGF-A splice isoforms [11].

## 3. Regulation of the Expression of the Oncogenic Splice Factor SRSF1

Thus, it is clear that the expression of splice factors and of their protein kinases varies significantly in different tissues and in cancer. Yet surprisingly little is known about the factors that regulate the expression of splice factors and splice factor kinases, perhaps because their involvement in pathological processes has only become apparent relatively recently. If SRSF1 is indeed an oncogenic splice factor, it is then very important to understand how its expression is regulated. Several groups have begun to address this question and growing evidence suggests that SRSF1 expression is regulated at multiple levels. 

Myc is one of the best studied oncogenes; it encodes a transcription factor that binds to DNA elements known as E-boxes. A ChIP on ChIP analysis using CpG arrays suggested a decade ago that Myc might interact with the SRSF1 promoter [18]. More recently, Das et al. found that Myc activates the transcription of SRSF1 through two E-boxes. They show that Myc-dependent SRSF1 upregulation resulted in changes in alternative splicing of known SRSF1 targets favouring the expression of splice isoforms that are consistent with an oncogenic phenotype. In contrast, the knockdown of SRSF1 reduces the oncogenic activity of Myc [19]. Furthermore, SRPK1, the protein kinase that promotes the entry of SRSF1 into the nucleus, is transcriptionally repressed by the Wilms tumour suppressor WT1 [11]. Thus, both a known oncogene (Myc) and tumour suppressor transcription factor (WT1) can regulate the expression of the oncogenic splice factor SRSF1 and of SRPK1, a protein kinase that regulates its localization. 

Several splice factors are themselves alternatively spliced to express functionally distinct isoforms. Some splice factors even bind to their own pre-mRNAs to autoregulate their expression—for example, the splice factor SRSF2 (previously known as SC35) favours the expression of splice isoforms that are unstable at the mRNA level [20]. SRSF1 also appears to bind to its own pre-mRNA in a similar autoregulatory loop. There are at least six splice isoforms of SRSF1; one expresses the full length splice factor, but the other five are unproductive. Unproductive transcripts are targeted by nonsense-mediated decay (NMD) due to the presence of premature stop codons. However, SRSF1 also has cytoplasmic activities—like many splice factors, it is multifunctional. The ability of SRSF1 to downregulate its own expression is to a large extent mediated at the level of the regulation of translation [21]. 

There is also evidence that SRSF1 expression is regulated by microRNAs. Leukemia/lymphoma-related factor (LRF) is an oncogenic transcription factor. A recent study shows that LRF represses the microRNAs miR-28 and miR-505 [22]. These two microRNAs target the 3′UTR of SRSF1 mRNA. Thus, a reduction in LRF results in higher levels of the microRNAs and therefore reduced SRSF1 expression. In turn, the reduction in SRSF1 levels can result in genomic instability, cell cycle arrest, and apoptosis. Furthermore, there is an alternative intron in SRSF1 in between two highly conserved elements in its 3′UTR. Skipping of this alternative intron appears to increase transcript stability, perhaps by removing a microRNA binding site [23]. 

In summary, SRSF1 expression can be controlled at multiple levels; transcriptionally, cotranscriptionally (through alternative splicing), and posttranscriptionally (through the regulation of translation and mRNA stability). In other words, there are several ways to fine tune the expression of oncogenic SRSF1. The ability of Myc to regulate SRSF1 and of WT1 to regulate SRPK1 expression is particularly interesting as these two transcription factors are associated with a wide range of cancers. The upregulation of SRSF1 in cancer could also occur through a failure of its autoregulation or through the inactivation of specific microRNAs. Undoubtedly, the regulation of other splice factors is also likely to be complex and multilayered. There are clearly many ways in which to perturb splice factor levels in cancer. 

## 4. Alternative Splicing and Multistage Carcinogenesis

Histopathologists have known for a long time that there are distinct phases in the development of cancer. In other words, the onset of cancer is generally thought to be a multistep process. The classical example to illustrate this phenomenon is the adenoma-carcinoma sequence in the development of colorectal cancer. Intestinal epithelial cells form a thin layer that is constantly being replaced. These epithelial cells are associated with the formation of carcinomas. 

The sequence of events that leads to malignant disease is thought to be as follows: normal epithelium changes to hyperplastic epithelium; this change is often associated with loss of function of the *APC* (adenomatous polyposis coli) gene. *APC* encodes a complex multidomain, multifunctional tumour suppressor. It is involved in the regulation of the cell-cycle, apoptosis, intercellular adhesion, and cytoskeletal architecture. Further changes alter the hyperplastic epithelium to early, intermediate, and late adenomas. These changes can be driven by several genes, including *K-Ras*. *Ras *genes encode GTPases that are involved in cell signalling associated with cellular proliferation. Adenomas can then develop into carcinomas that eventually acquire the ability to invade and metastasize. This latter change is often associated with the loss of function of the *TP53* gene. *TP53* is the widely studied “guardian of the genome” tumour suppressor transcription factor that regulates a multitude of processes including the cell cycle, apoptosis, and the response to DNA damage. All three genes (*APC*, *K-Ras*, and* TP53*) express splice isoforms whose functional properties are distinct and even antagonistic. Several other genes have been shown to be involved in the aetiology of colorectal cancer; for the purposes of this review, only these three are discussed. However, it is important to note that other genes involved in colorectal cancer are also alternative spliced in malignancy, giving rise to functionally distinct isoforms. Notable examples are the mismatch repair genes *MLH1* and *MSH2* [24].

Several alternative splice isoforms of APC have been described that result in proteins with molecular weights ranging from 90 to 300 kDa. De Rosa and colleagues examined a cohort of 24 patients suffering from familial adenomatous polyposis (FAP), each with a germline mutation in the *APC* gene, comparing them to 17 FAP patients without apparent *APC *mutations and 9 controls [25]. Using nested 5′ and 3′ primers, they identified nine novel transcripts. Three of these preserved the open reading frame. One of the transcripts contained an additional exon termed exon 1A; its inclusion leads to a premature stop codon in exon 2. The inclusion of exon 1A was found to be 3.5-fold higher in colorectal cancer versus normal mucosa. Transcripts that harbour premature stop codons are generally degraded by nonsense mediated decay (NMD)—this was shown to be the case when exon 1A is included. The potential significance is that greater inclusion of exon 1A could effectively result in less APC protein being expressed because less productive mRNAs are synthesised overall. Another group took a different approach and analysed an unusual FAP patient. This patient had a missense mutation in codon 640. Rather than necessarily altering the functional properties of the protein, the mutation was shown to interfere with a splicing enhancer motif bound by the oncogenic splice factor SRSF1 [26]. The consequences of the mutation were found to be the skipping of exon 14. This exon skipping is what gives rise to the nonfunctional APC, not the missense mutation *per se*. These studies illustrate two principles. The first is the need to consider the potential effects of intronic mutations on alternative splicing (these are often ignored); and the second is the need to examine the potential consequences of exonic mutations on alternative splicing because exons contain splicing enhancer and silencer sequences.

Two splice variants of K-Ras have been described, K-Ras 4A and 4B. They arise from two alternative versions of exon 4. Whereas the inclusion of exon 4A results in a proapoptotic K-Ras, the inclusion of exon 4B results in an antiapoptotic protein. Both are coexpressed in many tissues, but their ratio is altered in sporadic colorectal cancer favouring the antiapoptotic 4B isoform [27]. However, in mice, the expression of the 4A splice variant is not required for normal development despite its expression in a wide range of tissues [28]. It is nonetheless conceivable that mutations that favour the expression of 4B over 4A might augment the oncogenic properties of activated K-Ras. 

The *TP53* gene was thought for a long time to encode a single protein, but it is now abundantly clear that it is extensively alternatively spliced [29]. A remarkably complex series of splice isoforms have been described and several of these can regulate the transcriptional activity of p53 [30, 31]. P53 is also thought to suppress tumorigenesis by inducing senescence; this is facilitated by the splice isoform p53*β*. The expression of p53*β* is induced by the splice factor SRSF3 (previously known as SRp20). The knockdown of SRSF3 induces senescence in fibroblasts through a p53-mediated mechanism [32]. SRSF3 binds to a consensus binding site in the exon that is unique to p53*β*. To add to these findings, another study published earlier in the year in the same journal demonstrated that the downregulation of SRSF3 induces G1 cell cycle arrest and apoptosis in colon cancer cells [33]. However, in this case, the G1 arrest and proapoptotic mechanism were shown to be through a change in the alternative splicing of homeodomain-interacting protein kinase 2 (HIPK-2); the consequences of SRSF3 downregulation on p53 alternative splicing were not examined in detail. However, HIPK2 phosphorylates p53 at serine 46, favouring its proapoptotic properties [34]. Thus, SRSF3, like most splice factors, appears to be working in a coordinated way perhaps on groups of transcripts that are involved in related pathways. 

**Figure 2:**
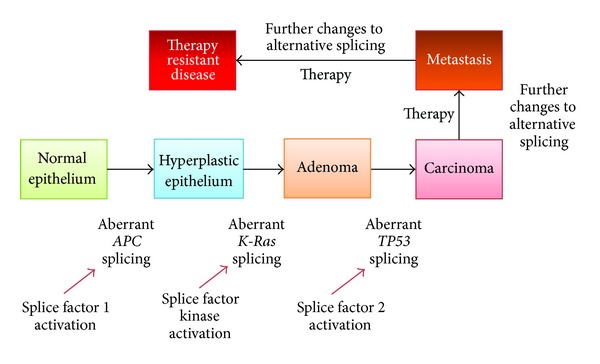
The adenoma-carcinoma sequence classically illustrates the multistage aetiology of colorectal cancer. Three genes frequently involved are *APC*, *K-Ras*, and *TP53*. This theoretical model suggests that the genetic lesions that drive the stages include changes that cause the inappropriate activity of oncogenic splice factors or splice factor kinases. The result is a significant change in the ratio of splice isoforms that drastically alter *APC*, *TP53*, and *K-Ras* function. Conventional treatments might cause selective pressures that drive further changes in the alternative splicing of key genes, leading to resistance to therapy.

Together, the observations on the alternative splicing of the *APC*, *K-Ras*, and *TP53* genes suggest that in the context of multistep carcinogenesis of colorectal cancer, genetic lesions that affect alternative splicing are likely to contribute significantly to the aetiology of disease (Figure 2). It is therefore not surprising that splice isoforms of these genes have been indicated as potential therapeutic targets in colorectal cancer [35]. 

## 5. Conclusion and Future Directions

The emergence of high-throughput next generation sequencing will make it possible to examine the transcriptomes of tumours in detail [36]. It will be possible to understand how aberrant alternative splicing can contribute to carcinogenesis and to the progression of disease. This is illustrated by a recent study in which normal epithelial cells were stressed with nicotine. The expression of 54,699 transcripts was examined; 173 were alternatively spliced in response to nicotine exposure. These transcripts encoded proteins associated with DNA recombination, replication, and repair [37]. It is therefore a good time to apply next generation sequencing technology to the study of alternative splicing in cancer systematically. However, a caveat to bear in mind is that alternative splicing patterns within primary, secondary tumour, and metastases are heterogenous, perhaps reflecting the differential expression and activity of splice factors in different parts of the tumour. Nonetheless, the power of next generation sequencing means that these complexities can also be addressed.

The number of publications that associate changes in alternative splicing with specific cancers has risen rapidly. It seems highly likely that alterations in the expression and activity of critical splice factors or of their modifiers (factor kinases and phosphatases) could result in a series of changes to the alternative splicing of several genes that together provide a growth or survival advantage to cancer cells. A systematic dysregulation of alternative splicing should therefore be considered yet another hallmark of cancer. 

Despite the growing evidence that alternative splicing plays an important role in cancer, the pharmaceutical sector has yet to exploit fully the therapeutic potential of manipulating alternative splicing. It is therefore arguably a very good time for funders to invest even more resources in basic research into the dysregulation of alternative splicing in cancer.
